# Effects of Diacutaneous Fibrolysis on Passive Neuromuscular Response and Mechanosensitivity in Athletes with Hamstring Shortening: A Randomized Controlled Trial

**DOI:** 10.3390/ijerph18126554

**Published:** 2021-06-18

**Authors:** Aida Cadellans-Arróniz, Carlos López-de-Celis, Albert Pérez-Bellmunt, Jacobo Rodríguez-Sanz, Luis Llurda-Almuzara, Vanessa González-Rueda, Pere Ramón Rodríguez-Rubio

**Affiliations:** 1 Faculty of Medicine and Health Sciences, Universitat Internacional de Catalunya, Carrer de Josep Trueta, Sant Cugat del Vallès, 08195 Barcelona, Spain; acadellans@uic.es (A.C.-A.); carlesldc@uic.es (C.L.-d.-C.); jrodriguezs@uic.es (J.R.-S.); lllurda@uic.es (L.L.-A.); vgonzalez@uic.es (V.G.-R.); prodriguez@uic.es (P.R.R.-R.); 2 ACTIUM Functional Anatomy Group, Carrer de Josep Trueta, Sant Cugat del Vallès, 08195 Barcelona, Spain; 3 Fundació Institut Universitari per a la Recerca a l’Atenció Primaria de Salut Jordi Gol i Gurina, 08007 Barcelona, Spain

**Keywords:** diacutaneous fibrolysis, neuromuscular response, tensiomyography, myotonometry, mechanosensibility, hamstring

## Abstract

Introduction. Diacutaneous Fibrolysis is defined as specific instrumental intervention to normalize function in the musculoskeletal system. It is considered a treatment method for the mechanical alterations of the locomotor system, and it is widely used in sports for therapeutic and preventive purposes. Despite the clinical benefits observed in different musculoskeletal conditions, the action mechanism of diacutaneous fibrolysis remains uncertain. There are no studies evaluating the neuromuscular response on the posterior muscular chain of the lower extremity in athletes, where overload, stiffness, and injury incidence are high. Objective. To evaluate the immediate, and 30 min post treatment effects of a single diacutaneous fibrolysis session on passive neuromuscular response and mechanosensitibity on hamstring and gluteus in athletes with shortening. Design. A randomized within participant clinical trial. Methods. Sixty-six athletes with hamstring shortening were included (PKE < 160). The lower limbs were randomized between the experimental limb and control limb, regardless of dominance. A single session of diacutaneous fibrolysis was applied to the posterior gluteus maximus, biceps femoris, and semitendinosus of the experimental lower limb whereas the control limb was not treated. Viscoelastic muscle properties (myotonometry), contractile muscle properties (tensomiography), and mechanosensitivity (algometry) were tested before treatment (T0), after treatment (T1), and 30 min post treatment (T2). Results. Regarding viscoelastic properties, in the intra-group analysis we found statistically significant differences in the experimental limb at T1, decreasing muscle stiffness in gluteus maximus (*p* < 0.042), in biceps femoris (*p* < 0.001) and in semitendinosus (*p* < 0.032). We also observed statistically significant differences in Tone decrease (*p* < 0.011) and relaxation increase (*p* < 0.001) in biceps femoris. At T2, the decrease in stiffness in all tested muscles was maintained (*p* < 0.05). There were statistically significant inter-groups differences in stiffness on gluteus (*p* < 0.048) and biceps femoris (*p* < 0.019) and in tone on biceps femoris (*p* < 0.009) compared to the control limb. For contractile properties, we only found statistically significant differences on maximal radial displacement (Dm) in gluteus, both control and experimental at T2 (*p* < 0.05) and in biceps femoris control (*p* < 0.030). No changes were found in the mechanosensitivity. Conclusions. A single session of diacutaneous fibrolysis produces changes in some parameters related to viscoelasticity properties of the biceps femoris and gluteus. There were no changes on contractile properties on semitendinosus. Only small changes on the contractile properties on the gluteus maximus and biceps femoris were found. No effect was found on the mechanosensitivity of the posterior chain muscles in athletes with hamstring shortening.

## 1. Introduction

Diacutaneous Fibrolysis (DF) is defined as “specific instrumental intervention to normalize function in the musculoskeletal system” [1]. It is considered a treatment method for the mechanical alterations of the neuromuscular system. It is applied by means of metallic hooks, ending in a spatula with beveled edges that seems to allow a deeper and more precise application, compared to the manual approach [2,3,4]. A recent systematic review and meta-analysis has been recently published reporting the effectiveness of DF on pain, range of motion, and function in musculoskeletal disorders, such as subacromial impingement syndrome, symptomatic patients with carpal tunnel syndrome, and chronic lateral epicondyalgia. However, none of the studies included focused on the lower limb [5]. The relationship between hamstring flexibility and injury has been widely investigated. Hamstring shortness has become an important risk factor for hamstring strain injury and loading changes in lower extremity biomechanics [6]. Moreover, hamstring shortness is neuromechanically characterized by an altered muscle length-tension relationship and muscle recruitment patterns. It is described that hamstring overactive-induced shortness changes sarcomere and viscoelastic properties [7,8]. Hamstring injuries are the most common muscle injury in athletes, involving mild alterations up to the complete loss of fiber organization, accounting for almost 30% of lower limb injury [6]. Specifically, the biceps femoris is the most commonly muscle injured (84%), followed by semimembranosus (12%) and the semitendinosus (4%) [9].

Despite the clinical benefits observed in different musculoskeletal conditions, the action mechanism of DF remains uncertain. It is not known whether this effect is a result of tension changes in the tissue (improvements on elasticity and stiffness) or due to reflex aspects, as suggested in other studies [10]. The Neuromuscular Response (NMR) is a set of biomechanical and viscoelastic properties of the myofascial tissue that prepare the muscle to perform a mechanical work as a result of muscular and nervous system function [11]. Myometry and tensiomyography are two tools used to assess NMR by analyzing different properties of muscle and fascial tissue [12]. Nevertheless, a study was recently published evaluating the DF effect on gastrocnemius NMR, in asymptomatic subjects, where a decrease in muscle tone and stiffness was found, maintaining its effects 30 min after the treatment [13].

In order to improve athletic performance, different soft tissue mobilizations techniques has been studied before in athletes [8,14,15]. DF is widely used in sports, for therapeutic and preventive purposes, but we have only found one study focuses on anterior knee pain, in athletes [16]. There are no studies evaluating its neuromuscular response effects in athletes with hamstring shortening, where overload, stiffness, and injury incidence are high [13,17,18,19]. Thus, the aim of this study is to evaluate the immediate and 30-min post treatment effects of a single diacutaneous fibrolysis session on viscoelastic and contractile muscle properties and mechanosensitibity on hamstring and gluteus in athletes with shortening.

## 2. Materials and Methods

### 2.1. Study Design

A randomized within participant (1:1) clinical trial was conducted. The study was registered at clinicaltrials.gov (study code: NCT04778293). The study protocol was approved by the local ethics committee (Comitè d’Ètica de Recerca—CER Universitat Internacional de Catalunya, study code: FIS-2020-04). The procedures followed were in accordance with the Declaration of Helsinki 1975, Fortaleza 2013. The study was conducted on the Universitat Internacional de Catalunya premises. All research was performed in accordance with Consort and TIDier guidelines/regulations. Informed consent was obtained from all participants before the intervention began.

### 2.2. Sample Size Calculation

The sample size was calculated based on, Alvarez-Diaz P. et al. findings [20]. The sample size was calculated using the GRANMO 7.12 program, accepting a α risk of 0.05, test two-side, a β risk of 0.20, with 3.4 of SD and 1.8 mm of difference for maximal radial displacement (Dm) of the tensiomigografphy for the biceps femoris. We estimated a follow-up loss of 15%, which would require 66 limbs per group.

### 2.3. Sample Selection Criteria

Sixty-six athletes from the Faculty of Medicine and Health Sciences of the Universitat Internacional de Catalunya were recruited between February to April 2021 to voluntarily participate.

The inclusion criteria comprised (1) being athletes over 18 years old, (2) being registered in a club or institution where they compete and practice sports on a regular basis, (3) having a hamstring shortening on both limbs (Passive Knee Extension test (PKE) < 160°) [21] and (4) signed the informed consent. The exclusion criteria were any contraindication related to diacutaneous fibrolysis such us poor skin or trophic condition, taking anticoagulants, suffering from any inflammatory process, or recent musculoskeletal lower limb injury (<6 month).

### 2.4. Randomization and Allocation

The limb assigned for diacutaneous fibrolysis treatment was randomized and the other limb was considered as the control. For the randomization process, an external evaluator did a randomization list prior to the recruitment of the athletes, with a computer program (www.random.org, accessed on 1 February 2021), that generated a list of sequential numbers (1 to 66). The evaluator was unaware of the group assignment.

### 2.5. Measurements

Viscoelastic properties were considered as the primary outcome whereas contractile properties and mechanosensitivity were considered as the secondary outcomes. The collection and recorded measurements were performed by a clinical research, who was blind to experimental/control limb assignment. The measurement instruments used were independent of the influence of the assessor. The outcomes were measured at the beginning of the study (T0), immediately after the DF intervention (T1), and 30 min after the DF intervention (T2), at gluteus, biceps femoris, and semitendinosus muscles, in the order in which they are presented below. All, measurements were performed in a prone position on a padded bench.

### 2.6. Outcomes

#### 2.6.1. Viscoelastic Properties

**Figure 1:**
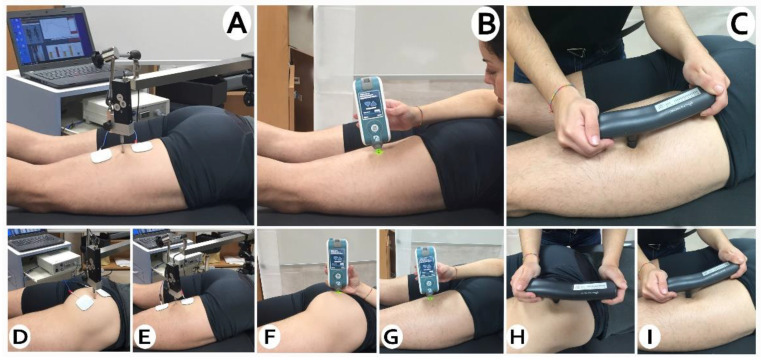
(**A**) Biceps femoris TMG. (**B**) Biceps femoris myotonometry. (**C**) Biceps femoris mechansonsibility. (**D**) Gluteus maximus TMG. (**E**) Semitendinosus TMG. (**F**) Gluteus maximus myotonometry. (**G**) Semitendinosus myotonometry. (**H**) Gluteus maximus mechansonsibility. (**I**) Semitendinosus mechansonsibility.

The tissue viscoelasticity were measured with the MyotonPro [ICC] = (0.80–0.93) [22,23] (MyotonPro, Myoton Ltd., Tallinn, Estonia). Three individual measurements with a recording interval of 1 s were performed and the mean values of stiffness (N/m), muscle tone (Hz), and relaxation (ms) were used for data analysis. The probe at the end of the device was placed perpendicular to the skin surface at the thickest point of the gluteus, biceps femoris, and semitedinosus muscles, selected by palpation, after a small voluntary contraction (Figure 1). Once this point was identified, it was marked with a permanent marker to ensure that the outcomes were taken in the same place for the subsequent measurements.

#### 2.6.2. Muscle Contractile Properties

Tensiomyograph (TMG) (TMG-BMC d.o.o., Ljubljana, Slovenia) was used for assessing the muscle contractile properties. It has good reliability for lower extremity muscles [24,25]. Percutanheously, it induces a muscle contraction by means of an electrical stimulus, which is detected by a digital transducer placed on the muscle belly to be evaluated (Figure 1). Self-adhesive electrodes (TMG electrodes, TMG-BMC d.o.o., Ljubljana, Slovenia) were situated equidistant to the measurement point, where the sensor was placed. The measurement points were the same as in the myotonometry. Electrical stimulation was applied via a TMG-100 System electrostimulator (TMG-BMC d.o.o., Ljubljana, Slovenia) with a pulse of 1 ms and an initial amplitude of 10 mA. In each trial, the amplitude was progressively increased in 10 mA increments, until there was no further increase in radial displacement and maximum stimulator power (100 mA).

All TMG parameters depend on the maximum radial displacement (Dm), which is the radial movement of the muscle belly after the application of the electrical stimulus, expressed in mm. Other parameter obtained with the TMG is contraction time (Tc), which is the time between 10% and 90% of Dm [9].

#### 2.6.3. Mechanosensibility

Mechanical tenderness was assessed in the same measurement points by applying progressive pressure until it reached 4 kg, by means of an algometer (handheld mechanical pressure algometer (Trigger Plus, Palpatronic, Hagen, Germany) [26]. Each participant was then asked to indicate whether he/she felt any pain sensation and to categorized it in a numerical scale from 0 to 10 [0—no pain, 10—maximum pain). This procedure has demonstrated good reliability [27,28].

### 2.7. Intervention

Participants received the DF intervention in one leg (experimental limb) previously randomized and no intervention on the opposite leg (control leg), regardless of dominance. A clinical researcher, with many years of experience in the technique, applied the DF. The study was conducted at the Functional Anatomy Laboratory of the Universitat Internacional de Catalunya, between February and April 2021.

**Figure 2:**
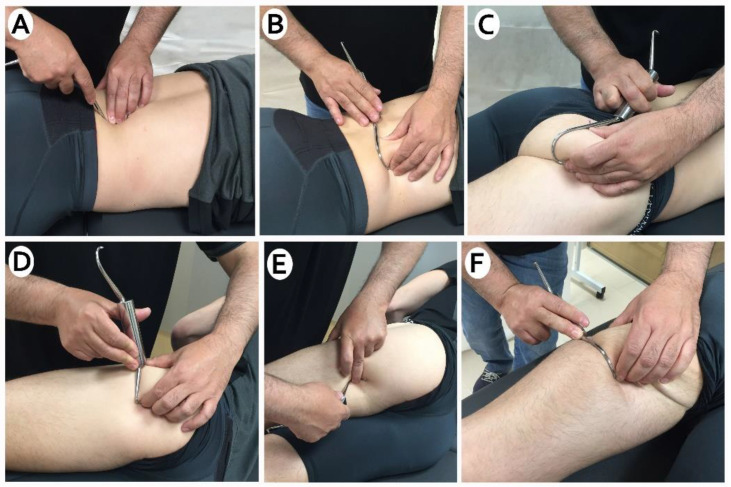
(**A**) Diacutaneous fibrolysis to paravertebral muscles, (**B**) Diacutaneous fibrolysis cuadratus lumbar, (**C**) Diacutaneous fibrolysis in gluteal area (**D**) Diacutaneous fibrolysis between vastus externus and biceps femoris. (**E**,**F**) Diacutaneos fibrolysis in hamstring area.

The experimental limb received DF treatment in the following musculature and intermuscular septa: quadratus lumbar, gluteus maixum, biceps femoris, and semitendinosus (Figure 2). With the patient lying in the prone position, the application was started in the lumbar paravertebral region, quadratus lumbar and iliac crest. The application continued on the gluteal and trochanteric region, and then by the posterior part of the tensor fascialis fascialis and vastus externus. It finished with the intermuscular septa between the vastus externus and biceps, biceps femoris, and semitendinosus. The time required for each diacutaneous session was about 10 min. The control limb did not receive any treatment.

The room temperature was controlled between 22 °C and 23 °C to avoid any alteration of the mechanical properties of the muscle [9]. DF technique was applied with the necessary pressure to encompass the structure to be moved. Brief rapid traction was applied in a transverse direction with the hook fixed to the skin and underlying soft tissues. No lotion was used.

### 2.8. Statistical Analysis

For statistical analysis, IBM SPSS Statistic 26.0 software was used. A descriptive analysis was carried out. For quantitative variables, mean and standard deviation were calculated. Frequencies were calculated for anthropometric qualitative variables. Normality distribution was assessed using Kolmogorov–Smirnov test, in order to know whether to use parametric or non-parametric tests.

Repeated measures ANOVA with a Bonferroni post hoc test was used for within-limbs changes over the measurement periods. Differences between limbs were observed using a paired *t*-test for those variables normally distributed, and a Wilcoxon test for those with non-normal distribution.

**Figure 3:**
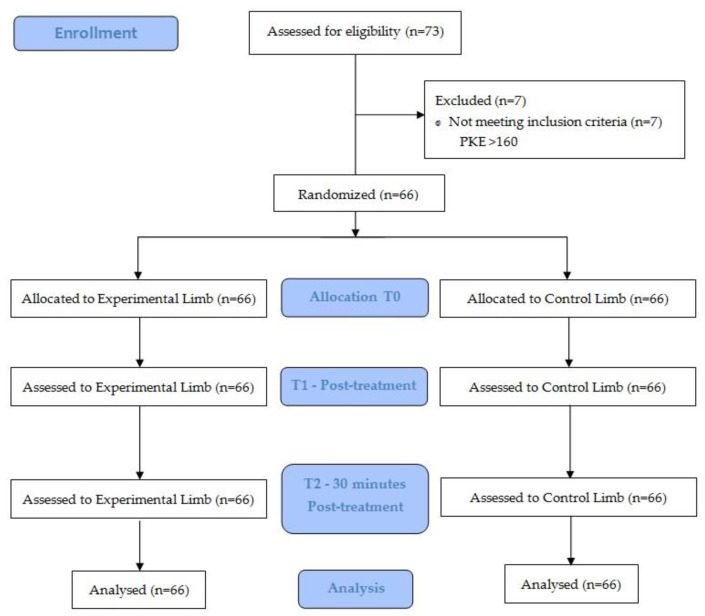
CONSORT. (Consolidated Standards of Reporting Trial) flow diagram.

Effect sizes were calculated using Cohen’s d coefficient [1]. An effect size > 0.8 was considered large; around 0.5, intermediate; and <0.2, small. Losses and exclusions after randomization are explained in Figure 3. Significance level was set at *p* < 0.05.

## 3. Results

Between February and April 2021, 73 volunteers were recruited (46 male, 27 female). Seven athletes, all of them females, were excluded (PKE test > 160°). The sample consisted of 66 athletes (66 experimental limbs and 66 control limbs). The mean age was 21.7 years (SD 3.5). There was no loss of follow-up (Figure 3).

The antrophometric characteristics of the sample are summarized in Table 1. No adverse or side effects were recorded. Football (14 athletes, 21.2%) was the most representative one across twenty registered sports. It was followed by Rugby with 8 athletes.

### 3.1. Viscoelastic Properties

In the intra-group analysis, we found statistically significant differences in the experimental limb at T1 in the gluteus maximus stiffness with a decrease of 10.16 N/m (*p* < 0.042; ES: 0.34). In biceps femoris we also observed statistically significant differences in Tone, with a decrease of 0.39 Hz (*p* < 0.011; ES: 0.23), in stiffness with 13.91 N/m (*p* < 0.001; ES: 0.37) and an increase in relaxation of 78 m/s (*p* < 0.001; ES: 0.29). A stiffness decrease of 9.72 (*p* < 0.035; ES: 0.20) on semitendinosus was also observed. At T2, there was a decrease in stiffness in all tested muscles: for Gluteus was of 9.09 N/m (*p* < 0.029; ES: 0.35), for biceps femoris of 6.50 N/m (*p* < 0.039; ES: 0.17) and for, Semitendinosus of 10.40 N/m (*p* < 0.042; ES: 0.22). A decrease in Gluteus tone of 0.21 N/m (*p* < 0.021; ES: 0.25) was maintained. Only the biceps femoris in T2 obtained a decrease in stiffness of 7.45 N/m (*p* < 0.037; ES: 0.20) in the control group.

In the intergroup analysis, we found statistically significant differences in the difference between T0–T2, in gluteus maximus, in stiffness (*p* < 0.048), in biceps femoris, in tone (*p* < 0.009) and in stiffness (*p* < 0.019). And between T0–T1, in the biceps femoris in relaxation (*p* < 0.045) (Table 2).

### 3.2. Contractile Properties

For this variable, we only found statistically significant differences in the intra-group analysis at T2. In the case of the experimental limbs, we found a decrease in the gluteus in Dm of 0.97 mm (*p* < 0.011; ES: 0.32). In the control limbs, we found a decrease in the gluteus at Dm 0.96 mm (*p* < 0.007; ES: 0.27) and a decrease in the biceps femoris at Dm of 0.60 mm (*p* < 0.030; ES: 0.22). There were no changes for the semitendinosus in the experimental and control limbs (Table 3).

In the intergroup analysis, we found no statistically significant differences between the experimental and control limbs.

### 3.3. Mechanosensibility

We did not find statistically significant differences in the intra-group analysis for the experimental and control limbs (*p* > 0.05) (Table 4), and neither for the intergroup analysis (Table 5).

## 4. Discussion

The present study aimed to assess the immediate and 30 min after effects of a single DF session on viscoelastic and contractile muscle properties and mechanosensibility on hamstring and gluteus maximus muscles, in athletes with hamstring shortening. Our results suggest that a single session of the DF generate changes in the tissue viscoelastic properties, without causing relevant changes in the contractile properties of the muscle. Furthermore, no changes in mechanosensitivity were observed.

Regarding the viscoelastic properties measured with myotonometry, the results indicate a decrease in the muscular stiffness and tone on biceps femoris for the experimental limb immediately after the DF treatment, compared to the control limb. In addition, there was observed a relaxation improvement on biceps femoris immediately after, which was maintained 30 min after the application of the technique. Immediately, stiffness improvements were also observed on gluteus maximus muscle.

With the results observed in this study, we suggest that the use of DF may be indicated in athletes to normalize neuromuscular response, related to viscoelastic properties, preserving their performance. In this sense, it should be noted that most of the variables have a moderate or small effect size. However, the fact that the intervention was only ten minutes long must be taken into account; we believe that a longer intervention could have modified these results. Several studies have associated hamstring injury risk with tone and stiffness increases in athletic population [10,13,29,30]. In this sense, a pre-competition DF intervention could be applied in cases of muscle stiffness or overload. The results obtained are in line with previous findings in similar populations using soft tissue mobilization techniques. A quasi-experimental clinical trial focusing on pre-competitive massage in athletes on the triceps surae muscle, also found a statistically significant reduction in tone and stiffness in the experimental [15].

Ikeda et al. [31] conducted a randomized control trial to evaluate the effects of instrumental techniques (IASTM) on plantar flexors and Achilles tendons in the ankle joint and muscle stiffness, assessed by elastosonogram. In contrast to our results, they found no change in muscle stiffness after the technique [31]. Nevertheless, the intervention they applied was based on a compressive tension and shearing to produce a longitudinal traction force on the compromised tissues around the edges of the instrument, which has an arc shape. In our opinion, the lack of changes in muscle stiffness could be explained by the characteristics of the technique and the instrument used. IASTIM may reach shallower planes than with hooks, which could reach deeper and more precise areas to mobilize them, transversely. Moreover, other issues that could explain the differences compared to our intervention is that the treatment lasted half as long (5 min) and it was applied on subjects without muscle shortening. In a descriptive-analytical study focusing on a single session of DF on gastrocnemius, improvements in stiffness and relaxation measured by myotonometry were found in healthy subjects, supporting the findings of the present study. However, they find a statistically significant increase in contractile properties, whereas contractile capacities do not appear to be modified in the present study [9].

To the best of our knowledge there are no previous studies evaluating contractile properties in athletes on hamstring. Our results indicate that there were no statistically significant differences between experimental and control limb, after the DF treatment. This finding differs from other studies where soft tissue mobilization techniques are used. Pérez-Bellmunt et al. [15] applied a pre-competition massage in athletes in order to assess the neuromuscular function. They found a significant increase in contraction time (Tc) measured by tensiomyography, suggesting that pre-competition massage could decrease the activation of this type II of fibers and thus increase the injury risk in high-speed sports. These differences may be because the massage directly targets the muscle belly, whereas the DF technique targets the intermuscular septa, applying a mainly transverse mobilization of the tissue.

Similar results were found by Leite et al. [32] when they evaluated the effects of DF on the gastrocnemius muscles in a recreational athletes population. They found improvements in the contractile properties of the muscle after DF, compared to the control group (sham DF). They suggest that the breakdown of adhesions would generate greater muscle excitability and force production, leading improvements in performance. Nevertheless, another study assessed the DF effects on gastrocnemius by TMG and only found statistically significant differences increasing the Dm after the application of technique, which was in line to their findings on tone and stiffness decrease, observed on MMT assessment [9]. The contractile properties by TMG data were also assessed by Macgregor et al. [33]. They conducted a randomized, crossover design in order to evaluate the neuromuscular effects of the instrument assist massage, by means of foam roller, on vastus lateralis (VL) and rectus femoris (RF) in active males. The contractile characteristics of RF were unaffected, as shown by TMG, although reduced muscle stiffness characteristics and increased contraction velocity were evident in VL on three days follow up [33].

Differing from other studies, our findings clearly point to that a single session of DF does not generate changes on the contractile properties of the studied musculature. Thus, it could be a therapeutic choice to normalize the viscoelastic properties of the hamstring and gluteus maximus without affecting the contractile properties, maintaining performance in sport. However, given the variability found in the literature involving the effects of tissue mobilization techniques on muscle contractile properties, measured by tensiomyography, we suggest investigating them in isolation. Other measurement tools that do not include mechanical aspects should be used, as appointed in other studies above [33]. Therefore, we believe that the results concerning the contractile properties in the present study cannot be conclusively confirmed.

The present study found a significant reduction in viscoelastic parameters in the biceps femoris, followed by the gluteus maximus muscle. These results could be due to an anatomical cause as the long head of the biceps has an anatomical continuity with the sacrotuberous ligament [34] and this ligament in turn is contiguous with the medial layer of the thoracolumbar fascia [35]. There is also a continuity between the outer layer of the thoracolumbar fascia and the gluteus maximus muscle [36]. There is also a fascial connection between this gluteus muscle and the proximal end of the biceps femoris [37]. Since these connections are produced by fascial tissue and not by muscle, it could explain why only changes in the viscoleastic parameters (myometry) and not in the contractile properties (tensiography) were observed.

The results obtained in the mechanosensitivity assessment indicates no statistically significant differences in the mechanosensibility applied to the different points assessed. These results are consistent with a recent systematic review that included three studies examining changes in lower extremity pain in healthy subjects undergoing IASTM [38]. Although our participants experienced muscle shortening, this condition is not associated with pain. As the baseline, baseline data showed very low values on the NRS scale it could explain the lack of improvements in mechanosensibility.

However, it should be noted that after the DF intervention, there was also no increase in tissue tenderness, as it has been reported previously in other IASTMs or manual therapy techniques aimed at normalizing the viscoelastic properties of tissue, such as deep transverse massage or dry needling [39].

### Limitations

This study has some limitations that could affect the generalizability of our results. Firstly, even though the effects were assessed 30 min after the application of the technique, there was no long-term follow-up. Secondly, in order to avoid external contamination of the outcomes assessed, only the effect of a single DF session was evaluated. Thus, we cannot know the cumulative effect of the technique, as it is usually clinically applied. Therefore, it is possible that the changes observed are minimal. Furthermore, we did not take into account the training cycle of the athletes; including those who were in the same phase (out or middle season) would have allowed us to obtain a more homogeneous sample. Also, unlike other clinical trials, the technique has not been applied as simulated, so we were not able to control for the possible placebo effect compared to the control limb. The skin stimulation by simulated DF is more superficial but has shown some beneficial effects in patients. Although it was not the main objective of the study, it would be interesting to evaluate its effect in comparison with the real technique. Finally, being an intra subject study allows a greater homogeneity of the groups, but we cannot discard a central effect involving effects on both limbs.

## 5. Conclusions

A single session of diacutaneous fibrolysis produces immediate improvements in the viscoelastic muscle properties (stiffness and tone) of the biceps femoris and Gluteus maximus. There were also smart changes on the contractile properties of the gluteus maximus and biceps femoris. There were no changes on contractile properties on semitendinosus. No effect was found on the mechanosensitivity of the posterior chain muscles in athletes with hamstring shortening.

## Figures and Tables

**Table 1:** Characteristics of the participants.

Clinical Features	Mean ± SD or *n* (%) (*n* = 66)
Age (years)	21.7 ± 3.5
Sex	
Men	46 (69.7%)
Women	20 (30.3%)
Height (cm)	175.5 ± 8.34
Weight (kg)	70 ± 11.89
BMI (kg/m^2^)	22.71 ± 2.86
Dominance	
Right	50 (75.8%)
Left	16 (24.2%)

Abbreviations: SD, Standard Deviation; *n*, number, %, percentage; cm, centimeters; kg, kilograms; m, meters.

**Table 2:** Intra-group viscoelastic properties analysis.

Variables		T0	T1	Difference T0–T1				T2	Difference T0–T2			
		Mean ± SD	Mean ± SD	Mean	95% CI	*p*	ES	Mean ± SD	Mean	95% CI	*p*	ES
Experimental Limbs												
Gluteus	Tone (Hz)	11.01 ± 0.84	10.75 ± 1.55	−0.26	[−0.657; 0.133]	0.325	0.21	10.81 ± 0.74	−0.21	[−0.388; −0.024]	0.021	0.25
	Stiffness (N/m)	159.70 ± 30.76	149.54 ± 29.07	−10.16	[−20.033; −0.286]	0.042	0.34	150.61 ± 19.80	−9.09	[−17.467; −0.715]	0.029	0.35
	Relaxation (m/s)	31.33 ± 4.09	31.79 ± 5.18	0.46	[−1.021; 1.938]	1.000	0.10	31.42 ± 4.59	0.08	[−1.340; 1.510]	1.000	0.02
Biceps Femoris	Tone (Hz)	15.85 ± 1.73	15.47 ± 1.56	−0.39	[−0.702; −0.072]	0.011	0.23	15.70 ± 1.53	−0.15	[−0.463; 0.268]	0.764	0.09
	Stiffness (N/m)	286.09 ± 39.19	272.18 ± 36.30	−13.91	[−19.417; −8.401]	0.001	0.37	279 59 ± 38.83	−6.50	[−12.751; −0.249]	0.039	0.17
	Relaxation (m/s)	18.98 ± 2.64	19.76 ± 2.81	0.78	[0.381; 1.180]	0.001	0.29	19.32 ± 2.70	0.34	[−0.032; 0.714]	0.084	0.13
Semitendinosus	Tone (Hz)	15.23 ± 1.89	15.06 ± 1.71	−0.17	[−0.510; 0.164]	0.638	0.09	15.22 ± 1.69	−0.02	[−0.375; 0.345]	1.000	0.01
	Stiffness (N/m)	269.52 ± 51.75	259.80 ±46.03	−9.72	[−18.917; −0.513]	0.035	0.20	259.12 ± 42.83	−10.40	[−20.521; −0.273]	0.042	0.22
	Relaxation (m/s)	19.62 ± 4.96	20.35 ± 4.06	0.74	[−0.066; 1.537]	0.083	0.16	20.12 ± 3.35	0.50	[−0.431; 1.440]	0.570	0.12
Control Limbs												
Gluteus	Tone (Hz)	10.95 ± 0.93	10.87 ± 0.84	−0.08	[−0.302; 0.138]	1.000	0.09	10.75 ± 0.76	−0.20	[−0.443; 0.052]	0.170	0.24
	Stiffness (N/m)	156.74 ± 24.99	154.21 ± 20.49	−2.53	[−9.443; 4.382]	1.000	0.11	150.48 ± 21.33	−6.26	[−13.765; 1.250]	0.134	0.27
	Relaxation (m/s)	31.67 ± 3.48	31.72 ± 3.10	0.05	[−0.777; 0.877]	1.000	0.02	31.65 ± 3.11	−0.02	[−0.880; 0.847]	1.000	0.01
Biceps Femoris	Tone (Hz)	15.96 ± 1.78	15.96 ± 1.72	0.00	[−0.306; 0.310]	1.000	0.00	15.72 ± 1.60	−0.24	[−0.544; 0.065]	0.174	0.19
	Stiffness (N/m)	289.33 ± 42.27	281.85 ± 42.40	−7.48	[−16.873; 1.903]	0.163	0.18	281.88 ± 36.10	−7.45	[−14.584; −0.325]	0.037	0.20
	Relaxation (m/s)	18.86 ± 2.95	19.25 ± 3.18	0.39	[−0.975; 0.194]	0.316	0.13	19.16 ± 2.79	0.30	[−0.169; 0.763]	0.367	0.10
Semitendinosus	Tone (Hz)	15.51 ± 2.13	15.33 ± 1.98	−0.18	[−0.551; 0.200]	0.764	0.09	15.48 ± 2.00	−0.03	[−0.438; −0.371]	1.000	0.02
	Stiffness (N/m)	273.11 ± 56.04	265.70 ± 49.10	−7.41	[−18.392; 3.574]	0.307	0.14	266.53 ± 49.04	−6.58	[−17.374; 4.222]	0.418	0.13
	Relaxation (m/s)	19.60 ± 4.28	19.83 ± 3.61	0.24	[−0.537; 1.014]	1.000	0.06	19.57 ± 3.83	−0.02	[−0.880; 0.834]	1.000	0.01

Abbreviations: SD, Standard Deviation; CI, Confidence interval; *p*, *p*-value; ES, Effect size; Hz, herzius; N/m, Newton/meter; m/s, meter/second.

**Table 3:** Intra-group contractile properties analysis.

Variables		T0	T1	Difference T0–T1				T2	Difference T0–T2			
		Mean ± SD	Mean ± SD	Mean	95% CI	*p*	ES	Mean ± SD	Mean	95% CI	*p*	ES
Experimental Limbs												
Gluteus	Tc (ms)	39.85 ± 16.74	43.35 ± 22.32	3.50	[−4.110; 11.112]	0.787	0.18	35.68 ± 12.43	−4.17	[−8.876; 0.536]	0.099	0.28
	Dm (mm)	5.41 ± 3.16	4.88 ± 3.14	−0.53	[−1.427; 0.361]	0.443	0.17	4.44 ± 2.86	−0.97	[1.760; 0.178]	0.011	0.32
Biceps Femoris	Tc (ms)	34.13 ± 14.29	32.26 ± 12.27	−1.87	[−5.772; 2.034]	0.731	0.14	32.16 ± 14.26	−1.97	[−6.230; 2.298]	0.784	0.14
	Dm (mm)	4.71 ± 2.88	4.61 ± 2.72	−0.10	[−0.816; 0.621]	1.000	0.04	4.46 ± 2.84	−0.24	[−0.916; 0.428]	1.000	0.09
Semitendinosus	Tc (ms)	40.32 ± 12.11	38.95 ± 12.40	−1.37	[−4.530; 1.794]	0.875	0.11	40.97 ± 12.05	0.65	[−2.431; 3.734]	1.000	0.05
	Dm (mm)	6.70 ± 2.80	6.43 ± 3.08	−0.27	[−0.805; 0.266]	0.663	0.09	6.37 ± 3.11	−0.34	[−0.954; 0.280]	0.554	0.11
Control Limbs												
Gluteus	Tc (ms)	44.03 ± 27.19	43.93 ± 25.93	−0.10	[−10.389; 10.186]	1.000	0.00	39.93 ± 16.10	−4.10	[−13.480; 5.284]	0.861	0.28
	Dm (mm)	6.24 ± 3.75	5.55 ± 3.50	−0.69	[−1.470; 0.098]	0.106	0.19	5.28 ± 3.32	−0.96	[−1.705; −0.212]	0.007	0.27
Biceps Femoris	Tc (ms)	34.63 ± 13.33	32.42 ± 14.17	−2.21	[−6.697; 2.278]	0.692	0.16	35.28 ± 16.85	0.65	[−4.760; 6.058]	1.000	0.04
	Dm (mm)	5.12 ± 2.83	4.44 ± 3.07	−0.67	[−1.453; 0.107]	0.113	0.23	4.51 ± 2.62	−0.60	[−1.162; −0.044]	0.030	0.22
Semitendinosus	Tc (ms)	41.36 ± 12.64	41.73 ± 12.05	0.38	[−3.340; 4.094]	1.000	0.03	42.81 ± 10.66	1.45	[−2.465; 5.369]	1.000	0.12
	Dm (mm)	6.83 ± 2.86	6.60 ± 2.94	−0.23	[−0.961; 0.507]	1.000	0.08	6.38 ± 3.02	−0.45	[−1.280; 0.377]	0.556	0.15

Abbreviations: SD, Standard Deviation; CI, Confidence interval; *p*, *p*-value; ES, Effect size; Tc, Contraction time; Dm, maximal displacement; ms, miliseconds; mm, milimeters.

**Table 4:** Intra-group mechanosensibility data analysis.

	T0	T1	Difference T0–T1				T2	Difference T0–T2			
	Mean ± SD	Mean ± SD	Mean	95% CI	*p*	ES	Mean ± SD	Mean	95% CI	*p*	ES
Experimental Limb											
Gluteus (NPRS 0–10)	1.17 ± 1.83	1.27 ± 1.85	0.11	[−0.160; 0.372]	0.992	0.05	1.21 ± 1.84	0.05	[−0.275; 0.366]	1.000	0.02
Biceps Femoris (NPRS 0–10)	1.02 ± 1.55	0.83 ± 1.63	−0.18	[−0.441; 0.078]	0.269	0.14	0.85 ± 1.56	−0.17	[−0.450; 0.117]	0.461	0.11
Semitendinosus (NPRS 0–10)	1.00 ± 1.36	0.94 ± 1.46	−0.06	[−0.391; 0.270]	1.000	0.04	0.92 ± 1.44	−0.08	[−0.445; 0.293]	1.000	0.06
Control Limb											
Gluteus (NPRS 0–10)	1.17 ± 1.67	1.20 ± 1.65	0.03	[−0.182; 0.242]	1.000	0.02	1.17 ± 1.79	−0.00	[−0.276; 0.276]	1.000	0.00
Biceps Femoris (NPRS 0–10)	1.05 ± 1.47	0.91 ± 1.43	−0.14	[−0.354; 0.082]	0.388	0.10	1.05 ± 1.65	−0.00	[−0.286; 0.286]	1.000	0.00
Semitendinosus (NPRS 0–10)	0.88 ± 1.23	0.85 ± 1.37	−0.03	[−0.311; 0.250]	1.000	0.02	0.89 ± 1.30	0.02	[−0.278; 0.308]	1.000	0.01

Abbreviations: SD. Standard Deviation; CI, Confidence interval; *p*, *p*-value; ES, Effect size; NPRS, numeric pain rating scale.

**Table 5:** Differences in inter-limbs data analysis.

Variable		Difference T0–T1			Difference T0–T2		
		Experimental Limbs	Control Limbs		Experimental Limbs	Control Limbs	
		Mean ± SD	Mean ± SD	*p*	Mean ± SD	Mean ± SD	*p*
Gluteus	Tone (Hz)	−0.26 ± 1.31	−0.08 ± 0.73	0.283	−0.21 ± 0.60	−0.20 ± 0.82	0.829
	Stiffness (N/m)	−10.16 ± 32.64	−2.53 ± 22.85	0.048	−9.09 ± 27.69	−6.26 ± 24.82	0.289
	Relaxation (m/s)	0.46 ± 4.89	0.05 ± 2.73	0.633	0.08 ± 4.71	−0.02 ± 2.85	0.246
	Tc (ms)	3.50 ± 25.16	−0.10 ± 34.01	0.466	−4.17 ± 15.56	−4.10 ± 31.02	0.699
	Dm (mm)	−0.53 ± 2.95	−0.69 ± 2.59	0.687	−0.97 ± 2.62	−0.96 ± 2.47	0.982 *
	MCS (NPRS 0–10)	0.11 ± 0.88	0.03 ± 0.70	0.553	0.05 ± 1.06	−0.00 ± 0.91	0.880
Biceps Femoris	Tone (Hz)	−0.39 ± 1.04	−0.00 ± 1.02	0.009	−0.15 ± 1.04	−0.24 ± 1.01	0.960
	Stiffness (N/m)	−13.91 ± 18.21	−7.48 ± 31.04	0.019	−6.50 ± 20.67	−7.45 ± 23.57	0.909
	Relaxation (m/s)	0.78 ± 1.32	0.39 ± 1.93	0.053	0.34 ± 1.23	−0.30 ± 1.54	0.045
	Tc (ms)	−1.87 ± 12.90	−2.21 ± 14.83	0.546	−1.97 ± 14.10	−0.65 ± 17.88	0.891
	Dm (mm)	−0.10 ±2.38	−0.67 ± 2.58	0.120	−0.24 ± 2.22	−0.60 ± 1.85	0.134
	MCS (NPRS 0–10)	−0.18 ± 0.86	−0.14 ± 0.72	0.365	−0.17 ± 0.94	−0.00 ± 0.94	0.534
Semitendinosus	Tone (Hz)	−0.17 ± 1.11	−0.18 ± 1.24	0.803	−0.02 ± 1.19	−0.03 ± 1.34	0.766
	Stiffness (N/m)	−9.72 ± 30.42	−7.41 ± 36.31	0.325	−10.40 ± 33.47	−6.58 ± 35.70	0.263
	Relaxation (m/s)	0.74 ± 2.65	0.24 ± 2.56	0.344	0.50 ± 3.09	−0.02 ± 2.83	0.114
	Tc (ms)	−1.37 ± 10.45	0.38 ± 12.29	0.502	0.65 ± 10.19	1.45 ± 12.95	0.771
	Dm (mm)	−0.27 ± 1.77	−0.23 ± 2.43	0.690	−0.34 ± 2.04	−0.45 ± 2.74	0.074 *
	MCS (NPRS 0–10)	−0.06 ± 1.09	−0.03 ± 0.93	0.742	−0.08 ± 1.22	0.02 ± 0.97	0.487

Abbreviations: SD. Standard Deviation; CI, Confidence interval; *p*, *p*-value; ES, Effect size; * paried student t test; Tc, Contraction time; Dm, maximal displacement; Hz, herzius; N/m, Newton/meter; ms, miliseconds; mm, milimeters; MCS, mechanosensitibity; NPRS, numeric pain rating scale.

## Data Availability

The data presented in this study are available on request from the corresponding author.
